# Driving Cells to the Desired State in a Bimodal Distribution through Manipulation of Internal Noise with Biologically Practicable Approaches

**DOI:** 10.1371/journal.pone.0167563

**Published:** 2016-12-02

**Authors:** Che-Chi Shu, Chen-Chao Yeh, Wun-Sin Jhang, Shih-Chiang Lo

**Affiliations:** Department of Chemical Engineering and Biotechnology, National Taipei University of Technology, Taipei City, Taiwan; King’s College London, UNITED KINGDOM

## Abstract

The stochastic nature of gene regulatory networks described by Chemical Master Equation (CME) leads to the distribution of proteins. A deterministic bistability is usually reflected as a bimodal distribution in stochastic simulations. Within a certain range of the parameter space, a bistable system exhibits two stable steady states, one at the low end and the other at the high end. Consequently, it appears to have a bimodal distribution with one sub-population (mode) around the low end and the other around the high end. In most cases, only one mode is favorable, and guiding cells to the desired state is valuable. Traditionally, the population was redistributed simply by adjusting the concentration of the inducer or the stimulator. However, this method has limitations; for example, the addition of stimulator cannot drive cells to the desired state in a common bistable system studied in this work. In fact, it pushes cells only to the undesired state. In addition, it causes a position shift of the modes, and this shift could be as large as the value of the mode itself. Such a side effect might damage coordination, and this problem can be avoided by applying a new method presented in this work. We illustrated how to manipulate the intensity of internal noise by using biologically practicable methods and utilized it to prompt the population to the desired mode. As we kept the deterministic behavior untouched, the aforementioned drawback was overcome. Remarkably, more than 96% of cells has been driven to the desired state. This method is genetically applicable to biological systems exhibiting a bimodal distribution resulting from bistability. Moreover, the reaction network studied in this work can easily be extended and applied to many other systems.

## Introduction

**Fig 1:**
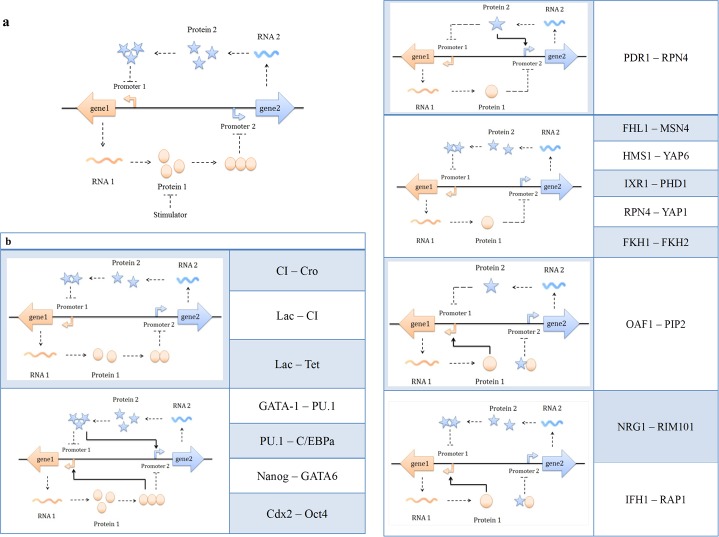
Reaction networks of systems with a genetic toggle switch **a)** One canonical motif of genetic toggle switches is composed of mutually inhibitory networks in which two proteins P1 and P2 inhibit the expression of each other. The subtle balance of two protein repressors decides the binary fate. The ON of P1 usually leads to the OFF of P2 and vice versa. **b)** Many regulatory systems share similar mutually inhibitory networks. This type of toggle switches can be found in bacteria, yeast, and mammalian cells.

Bistability has been studied greatly in synthetic biology as well as stochastic simulations [1–5]. A bistable system is featured with the presence of two stable steady states and the system usually behaves switch-like response between ON and OFF states. Normally, the ON state refers to the stable steady state with high gene expression and the OFF state to that with low gene expression. Bistability can be found in various biological systems, including nutritional uptake, viral infection, and quorum sensing [6–9]. A cell with bistable behavior stays at either one of two stable steady states; this binary response results from positive and/or negative feedback loops, which render nonlinearity to the reaction network[10]. Its outcome in the population is often a bimodal distribution with two modes reflecting two stable steady states, the ON and the OFF. The coexistence of bistability and a bimodal distribution has been reported in many publications [1, 4, 5, 11–13]. One of the most common types of bistable system, including the well-known system of bacteriophage λ[14], is the regulation of gene expression based on two promoters in a mutually inhibitory network. Notably, numerous systems have similar reaction networks (Fig 1) [11, 15–21]. Various species, from prokaryotes to eukaryotes, utilize this regulatory strategy and such a pattern is conserved from microorganisms to mammalian cells. The functions of these reaction networks are diverse, including uptake of nutrition, the response to stress, and the decision for the life cycle.

In the present work, the key components of the toggle switch are two repressible promoters (Fig 1A), each of which is inhibited by the repressor encoded in the other gene. Without the presence of repressors, the expression of the gene is constitutive. When one of the repressors prevails, the other gene becomes silent. In summary, the system enables the activation of only one promoter at a time; when one promoter is at ON state, the other promoter is at OFF state. Other systems sharing similar regulatory networks are listed in Fig 1B. They are slightly different from the system shown in Fig 1A but have the same basic framework. With further accounting for the positive feedback or the heterodimer as a repressor, the analysis on Fig 1A could be mostly applied to systems in Fig 1B. In this study, we examined the fundamental reaction network (Fig 1A) by both deterministic as well as stochastic models.

Stochasticity plays a vital role in most bistable systems. It is common for a cell to “jump” from one stable steady state to the other due to intracellular noise. Such noise-induced phenomena cause experimental studies under identical operational conditions to produce very different outcomes. Without a doubt, the jumping frequency is affected by the magnitude of the noise. The escalation of intracellular stochastic fluctuations was shown to shorten the mean first passage time [22] which quantifies the averaged time required for a cell to jump from one state to the other. The magnitude of stochastic fluctuations is indeed one of the most crucial factors; while fluctuations decline to zero, the stochastic models represent the deterministic trajectory.

It is practicable to manipulate the internal stochasticity of gene expression. Experimentally, Ozbudak demonstrated that the noise intensity of protein levels can be controlled by altering the rate of transcription and translation[23]. In his work, noise (defined as the standard deviation divided by the mean) was slightly affected by the rate of transcription but heavily influenced by the rate of translation. Thus, the magnitude of internal stochastic fluctuations can be adjusted without altering the mean value. Specifically, systems with distinct combinations of transcription and translation rates can have the same protein level from the perspective of deterministic models but have different noise intensity.

In living systems, driving cells into a specific mode is of high value. For example, the transfer of drug resistance in *Enterococcus faecalis* is featured with bistability[24]; the redistribution of cells to the OFF state can reduce the dissemination of drug resistance. For most of the systems in Fig 1B, only one stable steady state is desired and the other is unwanted. The bimodality results from stochasticity of gene expression; the value of each mode is the proximity to that of the corresponding stable steady state. A bistable curve occurs only within a certain range of stimulator concentrations. Once the concentration of the stimulator is higher or lower than the threshold values, the bistability vanishes and its consequence in population level is a unimodal distribution. Increasing or reducing the stimulator concentration changes the portion distributed in each mode and it is the conventional method of driving cells into the desired state. It functions effectively for many systems but inevitably has limitations. In this study, a novel strategy of utilizing internal stochasticity to redistribute cells is presented, and it overcomes some limitations of the conventional method.

## Models

### The reaction network

The gene regulatory network in which two proteins P1 and P2 inhibit the expression of each other (Fig 1A) appears to be a canonical motif of deciding the binary fate. This reaction network including two opposing fate-determining proteins is composed of a set of reactions in Table 1. The reactions first and second describe the transcription from active and inactive gene 1, respectively. The subscript 1 indicates gene 1. The reaction third shows the translation of P1. The reactions fourth and fifth are the processes of protein monomer to dimer, dimer to trimer. The reaction sixth depicts the binding of P1 trimer to promoter 2. When the trimer of P1 binds to promoter 2, the expression of gene 2 becomes inactive. The rest of reactions are similar but written for gene 2 and the binding of P2 trimer to promoter 1.

**Table 1:** The reactions of the system.

$DA_{1}\overset{k_{RA{}_{1}}}{\to }R_{1}+DA_{1}$	**(1)**
$DI_{1}\overset{k_{RI}{}_{{}_{1}}}{\to }R_{1}+DI_{1}$	**(2)**
$R_{1}\overset{k_{P{}_{1}}}{\to }P_{1}+R_{1}$	**(3)**
$2P_{1}\overset{k_{PP{}_{1}}}{\underset{k_{-PP{}_{1}}}{⇌}}PP_{1}$	**(4)**
$PP_{1}+P_{1}\overset{k_{PPP{}_{1}}}{\underset{k_{-PPP{}_{1}}}{⇌}}PPP_{1}$	**(5)**
$PPP_{1}+DA_{2}\overset{k_{DI{}_{2}}}{\underset{k_{-DI{}_{2}}}{⇌}}DI_{2}$	**(6)**
$DA_{2}\overset{k_{RA{}_{2}}}{\to }R_{2}+DA_{2}$	**(7)**
$DI_{2}\overset{k_{RI{}_{2}}}{\to }R_{2}+DI_{2}$	**(8)**
$R_{2}\overset{k_{P{}_{2}}}{\to }P_{2}+R_{2}$	**(9)**
$2P_{2}\overset{k_{PP{}_{2}}}{\underset{k_{-PP{}_{2}}}{⇌}}PP_{2}$	**(10)**
$PP_{2}+P_{2}\overset{k_{PPP{}_{2}}}{\underset{k_{-PPP{}_{2}}}{⇌}}PPP_{2}$	**(11)**
$PPP_{2}+DA_{1}\overset{k_{DI{}_{1}}}{\underset{k_{-DI{}_{1}}}{⇌}}DI_{1}$	**(12)**

### The Deterministic Model

**Fig 2:**
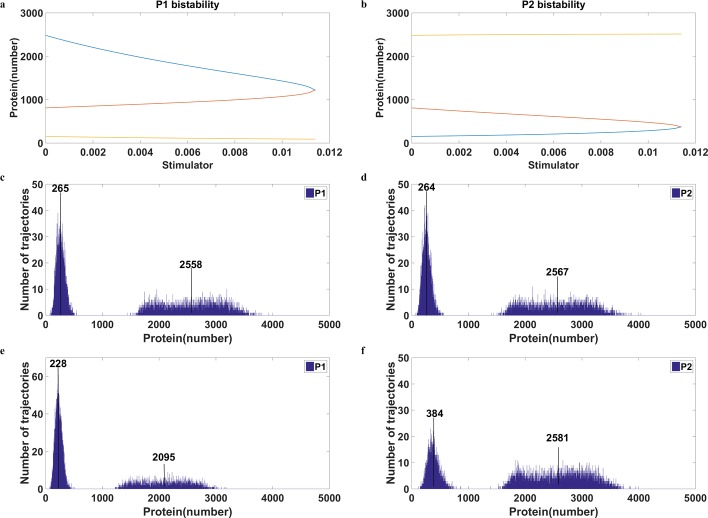
Bistable response and the corresponding bimodal distribution **a)** and **b)** show the bistable curves of P1 and P2, respectively. The x-axis represents the stimulator levels. As the stimulator increases, the ON state of P1 (cyan curve in **a)** shifts to a lower value and the OFF state of P2 (cyan curve in **b**) moves to a higher value. The yellow curve indicates the other stable steady state and the orange curve represents the unstable steady state. **c)** and **d)** show the bimodal distribution with no stimulator. **e)** and **f)** show the bimodal distribution with the stimulator of 0.003. The mean values of the protein level in each mode are indicated in the figure. The addition of the stimulator causes a shift of the ON state of P1 (from 2558 to 2095) as well as a shift of the OFF state of P2 (from 264 to 384).

Based on the reaction network shown in Table 1, we followed the law of mass action to formulate the deterministic model (Table 2 with the nomenclature listed in Table 3). The first and second equations describe the amount of DNA in active and inactive configurations, respectively. The third equation represents the concentration of RNA from gene 1. The 4^th^ to 6^th^ equations indicate the amount of P1 protein monomer, dimer, and trimer. The 7^th^ to 12^th^ equations are similar to 1^st^ to 6^th^ equations but they are written for gene 2. Note that the stimulator is not a variable in the above equations and it is assumed to directly act on P1 to speed up its degradation. The rate constant *k*_*r*_ in third equation is due to the stimulator and its value is set to zero except for the bistable figure in which the *k*_*r*_ is specified in the x-axis. The doubling time was assumed to be 30 min. The values of other parameters are listed in Table 4 which mainly adapted from literature [5, 25] (Text A in S1 File). This set of parameters has been used for all the simulations unless other values are specified in the text or figures. The ordinary differential equations, ODEs, in Table 2 were solved by ode15s in Matlab for deterministic dynamic behavior. The initial conditions of protein levels are given as the values of the unstable steady-state. The number of RNA is set to zero at the beginning and the DNA starts with two copies, one at the active configuration and the other at inactive conformation. The values of steady states shown in Fig 2 were calculated by fsolve in Matlab.

**Table 2:** The mass-action equations of the system.

$\frac{d\left[DA_{1}\right]}{dt}=k_{-DI{}_{1}}\left[DI_{1}\right]-k_{DI{}_{1}}\left[PPP_{2}\right]\left[DA_{1}\right]$	**(1)**
$\frac{d\left[DI_{1}\right]}{dt}=k_{DI{}_{1}}\left[PPP_{2}\right]\left[DA_{1}\right]-k_{-DI{}_{1}}\left[DI_{1}\right]$	**(2)**
$\frac{d\left[R_{1}\right]}{dt}=k_{RA{}_{1}}\left[DA_{1}\right]+k_{RI{}_{1}}\left[DI_{1}\right]-\left(k_{dR{}_{1}}+\mu \right)\left[R_{1}\right]$	**(3)**
$\frac{d\left[P_{1}\right]}{dt}=k_{P{}_{1}}\left[R_{1}\right]-2k_{PP_{1}}{\left[P_{1}\right]}^{2}+2k_{-PP{}_{1}}\left[PP_{1}\right]-k_{PPP_{1}}\left[PP_{1}\right]\left[P_{1}\right]+k_{-PPP_{1}}\left[PPP_{1}\right]-\left(k_{dP{}_{1}}+\mu +k_{r}\right)\left[P_{1}\right]$	**(4)**
$\frac{d\left[PP_{1}\right]}{dt}=k_{PP{}_{1}}{\left[P_{1}\right]}^{2}-k_{-PP{}_{1}}\left[PP_{1}\right]-k_{PPP{}_{1}}\left[PP_{1}\right]\left[P_{1}\right]+k_{-PPP{}_{1}}\left[PPP_{1}\right]$	**(5)**
$\frac{d\left[PPP_{1}\right]}{dt}=k_{PPP{}_{1}}\left[PP_{1}\right]\left[P_{1}\right]-k_{-PPP{}_{1}}\left[PPP_{1}\right]-k_{DI_{2}}\left[PPP_{1}\right]\left[DA_{2}\right]+k_{-DI{}_{2}}\left[DI_{2}\right]$	**(6)**
$\frac{d\left[DA_{2}\right]}{dt}=k_{-DI{}_{2}}\left[DI_{2}\right]-k_{DI{}_{2}}\left[PPP_{1}\right]\left[DA_{2}\right]$	**(7)**
$\frac{d\left[DI_{2}\right]}{dt}=k_{DI{}_{2}}\left[PPP_{1}\right]\left[DA_{2}\right]-k_{-DI{}_{2}}\left[DI_{2}\right]$	**(8)**
$\frac{d\left[R_{2}\right]}{dt}=k_{RA{}_{2}}\left[DA_{2}\right]+k_{RI{}_{2}}\left[DI_{2}\right]-\left(k_{dR{}_{2}}+\mu \right)\left[R_{2}\right]$	**(9)**
$\frac{d\left[P_{2}\right]}{dt}=k_{P_{2}}\left[R_{2}\right]-2k_{PP_{2}}{\left[P_{2}\right]}^{2}+2k_{-PP_{2}}\left[PP_{2}\right]-k_{PPP_{2}}\left[PP_{2}\right]\left[P_{2}\right]+k_{-PPP_{2}}\left[PPP_{2}\right]-\left(k_{dP_{2}}+\mu \right)\left[P_{2}\right]$	**(10)**
$\frac{d\left[PP_{2}\right]}{dt}=k_{PP{}_{2}}{\left[P_{2}\right]}^{2}-k_{-PP{}_{2}}\left[PP_{2}\right]-k_{PPP{}_{2}}\left[PP_{2}\right]\left[P_{2}\right]+k_{-PPP{}_{2}}\left[PPP_{2}\right]$	**(11)**
$\frac{d\left[PPP_{2}\right]}{dt}=k_{PPP{}_{2}}\left[PP_{2}\right]\left[P_{2}\right]-k_{-PPP{}_{2}}\left[PPP_{2}\right]-k_{DI{}_{1}}\left[PPP_{2}\right]\left[DA_{1}\right]+k_{-DI{}_{1}}\left[DI_{1}\right]$	**(12)**

**Table 3:** Nomenclature of the variables.

**Annotation**	**Description**
*DA*_*1*_	DNA of gene 1 in active formation
*DI*_*1*_	DNA of gene 1 in inactive formation
*R*_*1*_	RNA from gene 1
*P*_*1*_	Protein encoded in gene 1
*PP*_*1*_	P_1_ dimer
*PPP*_*1*_	P_1_ trimer
*DA*_*2*_	DNA of gene 2 in active formation
*DI*_*2*_	DNA of gene 2 in inactive formation
*R*_*2*_	RNA from gene 2
*P*_*2*_	Protein encoded in gene 2
*PP*_*2*_	P_2_ dimer
*PPP*_*2*_	P_2_ trimer

**Table 4:** The values of parameters.

**Parameter**	**Description**	**Value**	**Units**
$k_{RA{}_{1}}$	Transcription rate constant of active gene 1	2.1x 10^−1^	S^-1^
$k_{RI{}_{1}}$	Transcription rate constant of inactive gene 1	10^−2^	S^-1^
$k_{P{}_{1}}$	Translation rate constant of P_1_	10^−1^	S^-1^
$k_{PP_{1}}$	Rate constant of forming P_1_ dimer	10^−5^	nM^-1^S^-1^
$k_{-PP{}_{1}}$	Dissociation rate constant of P_1_ dimer	10^−1^	S^-1^
$k_{PPP_{1}}$	Rate constant of forming P_1_ trimer	10^−5^	nM^-1^S^-1^
$k_{-PPP_{1}}$	Dissociation rate constant of P_1_ trimer	10^−1^	S^-1^
$k_{DI_{2}}$	Binding rate constant of P_1_ trimer to promoter 2	10^−1^	nM^-1^S^-1^
$k_{-DI{}_{2}}$	Dissociation rate constant of P_1_ trimer and promoter 2	1	S^-1^
$k_{RA{}_{2}}$	Transcription rate constant of active gene 2	2.1x 10^−1^	S^-1^
$k_{RI{}_{2}}$	Transcription rate constant of inactive gene 2	10^−2^	S^-1^
$k_{P_{2}}$	Translation rate constant of P_2_	10^−1^	S^-1^
$k_{PP_{2}}$	Rate constant of forming P_2_ dimer	10^−5^	nM^-1^S^-1^
$k_{-PP_{2}}$	Dissociation rate constant of P_2_ dimer	10^−1^	S^-1^
$k_{PPP_{2}}$	Rate constant of forming P_2_ trimer	10^−5^	nM^-1^S^-1^
$k_{-PPP_{2}}$	Dissociation rate constant of P_2_ trimer	10^−1^	S^-1^
$k_{DI{}_{1}}$	Binding rate constant of P_2_ trimer to promoter 1	10^−1^	nM^-1^S^-1^
$k_{-DI{}_{1}}$	Dissociation rate constant of P_2_ trimer and promoter 1	1	S^-1^
$k_{dR{}_{1}}$	Degradation constant of R_1_	6.15x10^-4^	S^-1^
$k_{dP{}_{1}}$	Degradation constant of P_1_	1.63x10^-2^	S^-1^
$k_{dR{}_{2}}$	Degradation constant of R_2_	6.15x10^-4^	S^-1^
$k_{dP_{2}}$	Degradation constant of P_2_	1.63x10^-2^	S^-1^
*k*_*r*_	Degradation caused by stimulator		S^-1^
*μ*	Specific growth rate	3.85x10^-4^	S^-1^
*k*_*R*1_	Transcription rate constant of *R*_*1*_ from *Di*	1	S^-1^
*k*_*Ri*_	Transcription rate constant of *R*_*i*_ from *Di*	1	S^-1^
*k*_*d Ri*_	Degradation rate constant of *R*_*i*_	10^−3^	S^-1^
*k*_*X*_	Reaction rate constant of *R*_*i*_ and *R*_*i*_ interaction	10^−1^	nM^-1^S^-1^

### The Stochastic Model

**Fig 3:**
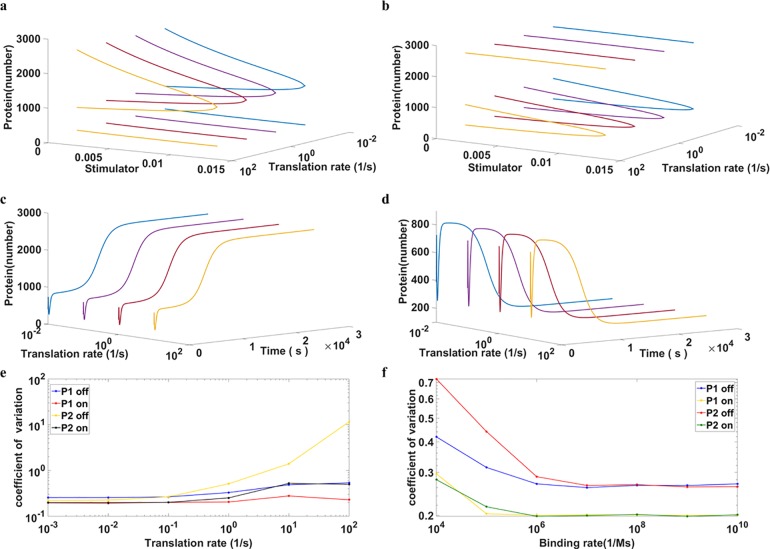
The change of noise intensity shows no effect on the deterministic response In panels **a)-e)**, all parameters are listed in Table 4 except the transcription and translation rate of gene 2. The translation rate of P2 is specified in the axis. The change of the translation rate of P2 is compensated by its upstream transcription rate so that the protein level remains constant. **a)** and **b)** are the bistable curves of P1 and P2, respectively. Different combinations of transcription and translation rates lead to the same deterministic steady-state behavior. **c)** and **d)** show dynamic responses of P1 and P2, respectively. Because the protein levels are the same for different translation rates, all dynamic responses of proteins are indistinguishable. **e)** illustrates the noise in each mode with different translation rates. The translation rates affect the intensity of noise. The higher the translation rate of P2 is, the greater the intensity of noise. Moreover, not only noise intensity of P2 but also that of P1 is influenced by the translation rate of P2. **f)** shows noise intensity changes in response to the rates of DNA-protein interaction. For this panel, all parameters are in Table 4, except for the binding and dissociating rate of P1 trimer binding to DNA. The binding rates are shown in the x-axis. The equilibrium constant is fixed so that the protein level is unchanged.

The mechanism of fate determination, in reality, is much more complicated than the description of the deterministic model. To clarify how multiplicative noise induces switching from one state to another, we then performed stochastic simulations. The Chemical Master Equation (CME) based on the reaction network shown in Table 1 is simulated by Stochastic Simulation Algorithm (SSA)[26] with software SynBioSS[27]. The cell volume is assumed 10^−15^ liter. The amount of trajectories computed were ten thousand and the raw data was then processed by Matlab. As shown in Fig 3C and 3D, about twelve thousand seconds are required for the system to reach the deterministic steady-state. In stochastic simulations, all results were collected from ten thousand trajectories at thirty thousand seconds; to ensure the stationary distribution was reached, the results from thirty thousand and sixty thousand seconds are compared and no significant difference of the distribution was found (Text B in S1 File). The number of cells in each model was calculated by separated cells into two groups; cells with protein concentration higher than unstable steady state were counted as ON state, otherwise OFF state. The standard deviation of each mode (Fig 3E and 3F) is then also calculated.

### The Influence of RNA-RNA interaction

The RNA-RNA interaction was conducted by producing RNAi from synthetic DNA. The binding of RNAi formed double-strand RNA and it is assumed that this dsRNA is very unstable. The loss of mRNA due to RNAi was compensated by more mRNA from the synthetic DNA so the protein level of steady states kept untouched. Based on the reaction network in Table 1, we further accounted for the following reactions.

$$D_{i}\overset{k_{Ri}}{\to }R_{i}+D_{i}$$ (1)

$$D_{i}\overset{k_{R1}}{\to }R_{1}+D_{i}$$ (2)

$$R_{i}+R_{1}\overset{k_{X}}{\to }\emptyset$$ (3)

where *R*_*i*_ is the RNA interacting with *R*_1_; *D*_*i*_ is the synthetic DNA producing both *R*_*i*_ and *R*_1_. The ∅ in Eq (3) represents degradation. The Eqs (1) and (2) are the transcription of RNAi and mRNA of gene 1 from synthetic DNA. After RNAi binding to mRNA of gene 1, the complex got degraded.

According to Eqs (1)–(3), the 3^rd^ equation of Table 2 became the equation below.

$$\frac{d\left[R_{1}\right]}{dt}=k_{RA{}_{1}}\left[DA_{1}\right]+k_{RI{}_{1}}\left[DI_{1}\right]+k_{R1}\left[D_{i}\right]-k_{X}\left[R_{i}\right]\left[R_{1}\right]-\left(k_{dR{}_{1}}+\mu \right)\left[R_{1}\right]$$ (4)

In addition, one more equation written for *R*_*i*_ is needed.

$$\frac{d\left[R_{i}\right]}{dt}=k_{Ri}\left[D_{i}\right]-k_{X}\left[R_{i}\right]\left[R_{1}\right]-\left(k_{d}{}_{Ri}+\mu \right)\left[R_{i}\right]$$ (5)

The values of parameters of Eqs (4) and (5) are all listed in Table 4.

## Results

Decisions of a cell are implemented through coordinating interactions of intracellular species. Fig 1A depicts the sufficient structure for a regulatory network to exhibit bistability. As mentioned in the Introduction, this framework represents key components of many biological systems (Fig 1B); with slight modifications, the conclusions of this study is applicable to numerous systems. The system examined in this study is composed of two promoters, two genes, and two repressors. Protein 1 (P1 in Fig 1A), encoded by gene 1 under the control of promoter 1, is the repressor of promoter 2. Similarly, gene 2 encodes protein 2 (P2) which represses promoter 1. The two promoters mutually inhibit each other and grant bistability to the system (Fig 1A).

### The Addition of Stimulator Alters the Position of the Peak

The conventional strategy of adjusting the ratio of the population dwelling in each mode is the addition of stimulator. For the bistable system shown in Fig 2A, raising the stimulator level increases the ratio of the OFF state of P1. With higher concentrations of the stimulator, more cells are in the OFF state. Finally, no cells remain in the ON state of P1 when the concentrations of the stimulator are higher than a critical value (0.0114 in Fig 2A) and consequently the entire cell population is in the OFF state (data not shown).

The method of using the stimulator to control the ratio of two modes is intuitive and efficient but entails limitations and side effects. Such a method is associated with two major problems. First, it is impossible to move more cells to the ON state of P1 in a system with no stimulator at the beginning because the concentration of the stimulator cannot be reduced further to a negative value. Second, the value of the mode is inevitably altered when the stimulator is added.

The steady state values of P1 and P2 with different levels of the stimulator were calculated by a deterministic model. The bistable curves in Fig 2A and 2B represent the steady-state protein levels of P1 and P2, respectively. The two stable steady states, cyan and yellow curves, correspond to the ON state and OFF state of P1. The ON state of P1 (cyan curve in Fig 2A) leads to the OFF state of P2 (cyan curve in Fig 2B) and vice versa. The unstable steady state shown by the orange curve in the middle (Fig 2A and 2B) cannot be experimentally maintained because of the stochastic gene expression. Also due to random fluctuations, the spontaneous switches of P1 from OFF (yellow curve in Fig 2A) to ON or ON to OFF becomes possible. Therefore, the system performs bimodal distribution.

The bimodal distributions with a stimulator concentration of zero are illustrated in Fig 2C and 2D for P1 and P2, respectively. With an increase in the stimulator, the system favors the OFF state of P1 and the ON state of P2. However, this rearrangement of the cellular population notably engenders the positional migration of modes. In Fig 2, the number above the mode indicates the mean value of protein levels in that mode. The mean of P1 at the ON without the stimulator was 2558 (Fig 2C), but this value became 2095 at a stimulator concentration of 0.003 (Fig 2E and 2F); a nearly 20% difference in the P1 level was observed. According to the bistable curves of the deterministic model (Fig 2A), the mode of the ON state could migrate all the way down to 1244 when the concentration of the simulator became 0.0113. The decrease from 2558 to 1244 constitutes a greater than onefold change.

### Manipulating the Stochasticity Showed No Effect on Deterministic Behaviors

The main regulatory strategy of gene expression is the transcriptional regulation of which protein (or peptide) interacting with DNA is one common pattern. The regulatory proteins in this study are P1 and P2. Although stochastic fluctuations are ubiquitous, the noise intensity of the protein level can be genetically modified. The rates of the transcription and the translation can be carefully chosen such that the noise intensity varies among combinations but protein levels remain fixed. Specifically, there is no difference of the deterministic bistable curves for different combinations. Moreover, the dynamic response of all variables except mRNA should be the same and the incremental rate of translation should merely cause higher noise intensity.

The noise generated from the translation is usually much higher than that generated from transcription, and enhancing translational efficiency escalates the intensity of the noise in protein levels. To maintain the same protein level, the rates of transcription were adjusted to compensate for variability in rates of translation (Text C in S1 File); the bistable curves were identical, as shown in Fig 3A and 3B for P1 and P2, respectively. These results imply that the steady-state behaviors of proteins are identical from the aspect of deterministic model regardless of translation rates. Intriguingly, the dynamic response of proteins also shows no difference for various translation rates (Fig 3C and 3D). From these observations, we concluded that the system, except for the expression level of RNA, is identical. Note that mRNA itself is not involved in regulatory processes.

Although the deterministic model is not capable of distinguishing differences in noise intensity, such differences can be captured by the stochastic model. The coefficient of variation of protein levels with several translation rates of P2 is shown in Fig 3E. Note that the coefficient of variation for the whole population is irrelevant because it is too sensitive to the allocation of the population; the maximum value of variance would occur while cells are equally divided into two modes (Text D in S1 File and S1 Fig). Instead, the coefficient of variation for individual modes is appropriate as it reflects the noise intensity in each mode. As expected, the OFF mode of P2 shows huge noise under the circumstance of a high translation rate of P2. The noise of P1 is much lower than that of P2 because P2 directly receives the noise from its own translation and then P1 inherits the noise from P2. One feature of the system is that the OFF state has higher noise than that of the ON state for both P1 and P2.

It is also possible to manipulate noise intensity with subtle adjustment of the protein-DNA binding and dissociating rates while keeping the equilibrium constant untouched. In particular, the fold change of the rate constants of dissociation is the same as that of the binding rate. Thus, the deterministic steady states are identical for all cases. The binding rate listed in Table 4 is for all other simulations and the value is 10^−1^(1/s nM) or 10^8^ (1/s M). When the binding rate was increased to 10^9^ or 10^10^, there was only a small change in noise intensity as shown in Fig 3F. This outcome was expected because the noise produced from fast reactions is averaged. Conversely, with a decrease of the binding rate, noise from protein-DNA interaction became influential and its effect on the protein level became noticeable (Fig 3F). Note that such an increment in noise is much smaller than that in the previous outcome (Fig 3E). A plausible explanation is that transcription and translation buffered the noise produced by protein-DNA interaction.

### Increasing the Ratio of Cells at On State by Manipulating Intracellular Stochasticity

**Fig 4:**
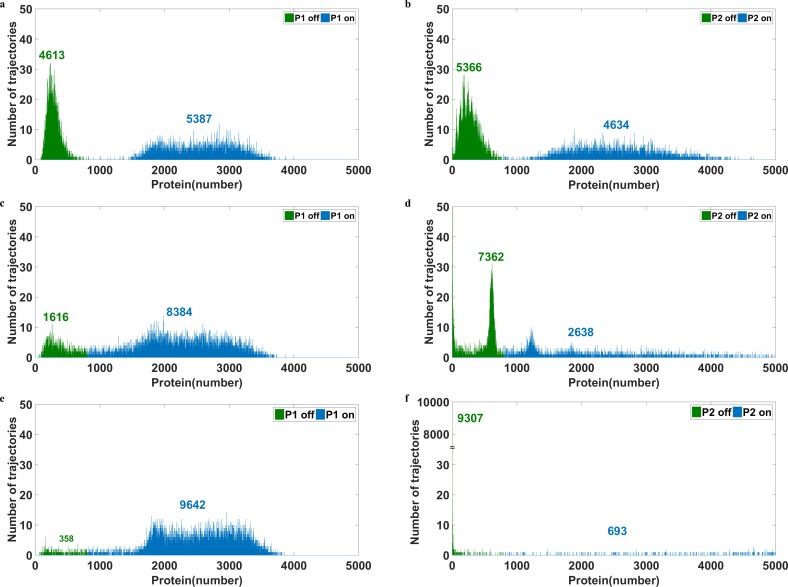
Manipulation of noise intensity leads cells to the desired state All parameters are listed in Table 4 except the transcription and translation rate of gene 2. The stimulator level is zero. The number above the mode indicates the cell number within the mode. Blue represents the ON state and green for the OFF state. Panels **a)** and **b)** show the bimodal distributions of P1 and P2 with the translation rate of P2 as 1(1/s). **c)** illustrates the possibility of guiding cells to the desired state by controlling the internal stochasticity of gene expression with the translation rate of P2 being 10 (1/s); more than 80% of cells moved to the ON state of P1. **d)** shows the distribution of P2. **e)** and **f)** show the bimodal distributions of P1 and P2, respectively, with the translation rate of P2 as 100 (1/s). Remarkably, more than 95% of cells were guided to the ON state of P1.

**Fig 5:**
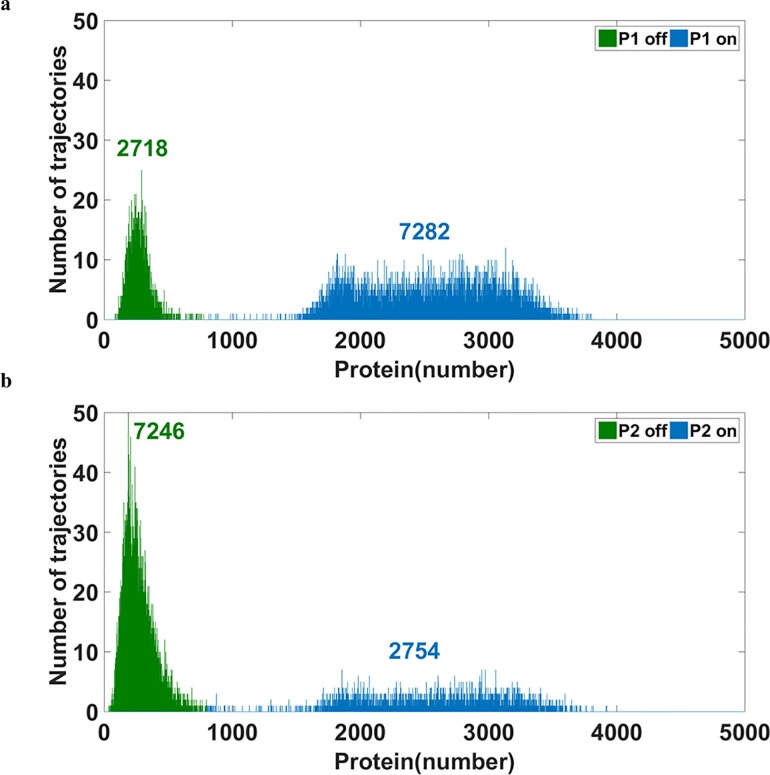
Influence of altering the reaction rate of protein-DNA binding The number above the mode indicates the number of cells in the mode. **a)** and **b)** represent the distributions of P1 and P2, respectively, with the reaction rate constant of P1 trimer binding to DNA being 10^5^ (1/s M). Approximately 72% of cells were in the ON state of P1 though there is only a slightly increment of noise intensity (Fig 3F).

**Fig 6:**
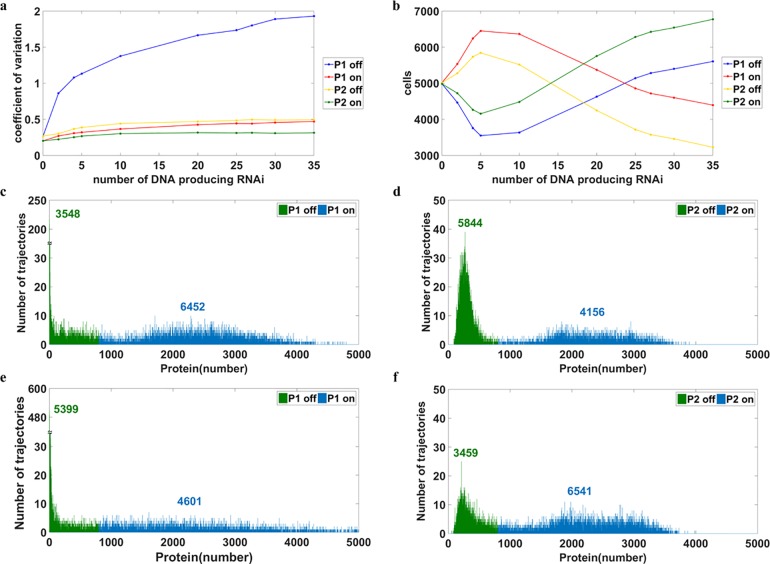
Effect of RNAi on noise intensity and the allocation of cells in the bimodal distribution. The number above the mode indicates the number of cells in the mode. **a)** illustrates how noise intensity changes as a response to the copy number of DNA producing RNAi. The OFF state of P1 has a relatively large amount of noise because the RNAi acted at mRNA of gene 1. **b)** provides the information of cell number at each stable steady state. Intriguingly, the increment of noise does not always drive cells to the desired state. **c)** and **d)** show the distributions of P1 and P2 for 5 copies of DNA which produce RNAi **e)** and **f)** show the bimodal distribution of P1 and P2 for 30 copies of DNA which produces RNAi.

The ON state of P1 can not be attained by conventional methods because the addition of a stimulator drives the population to the OFF state. However, it can be achieved by altering the noise intensity of the protein levels. In Figs 4–6, all the simulations were performed with no stimulator and the difference of the distributions resulted from distinct noise intensities. The number above the mode indicates the number of cells in that mode. Intriguingly, by using a higher translation rate of P2, 1 (1/s), to escalate random fluctuations, cells were driven to the ON state of P1 (Fig 4A). Consequently, the number of cells in the OFF state of P2 increased (Fig 4B). The distributions obtained by translation rate of P2 as 0.1 (1/s), which is the original parameter listed in Table 4, were shown in Fig 2C and 2D; there are 5017 cells at the ON state of P1 and 4984 cells at ON of P2. No doubt, the number of the cells at the OFF state of P1 is 4983 and that of P2 is 5016 because the total trajectories simulated are 10000. In a comparison of Fig 4A and 4B to Fig 2C and 2D, only about 3% increase in the ON state of P1 was observed. To drive more cells to the ON state of P1, the noise intensity of P2 protein was further increased (but the protein level was still kept the same by adjusting the transcription rate). As the translation rate of P2 reached 10 (1/s) (Fig 4C and 4D), the bimodal distribution of P2 was compromised and its shape collapsed; the noise of P2 was too large for the system to maintain the bimodal distribution. By contrast, much less noise is associated with P1, and the bimodal shape remained with most cells migrating to the ON state; 8384 cells were moved to the ON state of P1 (Fig 4C), because of random fluctuations. Moreover, when we increased the noise further with the translation rate of P2 to 100 (1/s), 9642 cells moved to the ON state of P1 (Fig 4E), and most of the cells had very low expression of P2 (Fig 4F). Fig 4 shows the possibility of utilizing stochasticity to prompt cells to the desired state. Although the above simulation was conducted with an initial condition of unstable steady states, the same results were also obtained from an initial condition of OFF stable steady state of P1 (S2 Fig). The mRNA did not directly participate in regulatory processes and its distributions were shown in S3 Fig. This new approach provides a feasible path for a task which is otherwise difficult to achieve with the addition of stimulator. This approach also enables driving cells back to the OFF state of P1 by changing the stochasticity of protein levels in the opposite direction (S4 Fig).

Other methods of manipulating noise intensity can also be utilized to guide cells to the desired state. The outcome of changing rates of protein-DNA binding reaction was illustrated in Fig 5. Approximately 72% of cells moved to the ON state of P1 (Fig 5A) or the OFF state of P2 (Fig 5B) for the case which the rate constant of P1 trimer binding to DNA was 10^5^ (1/s M). Further increases in noise did not move more cells into the desired state (S5 Fig). Although it was less than 96.4% obtained from the previous method, the distribution was much narrow in both modes. For systems, low noise intensity is usually better than high noise intensity. The later may impede coordination, which is considered a critical characteristic of multiple steady states [28].

Noise intensity can also be altered through RNA-RNA interaction, but compensation is required for keeping steady states intact (refer to Models for details). In brief, RNAi was added to the system to cause RNA-RNA interaction and escalated the intensity of noise at the protein level. This method substantially increased noise and provided an opportunity to examine its effect on the system (Fig 6A and 6B). For the regions with relatively low noise (the regions with the copy number of DNA producing RNAi lower than 5 in Fig 6A and 6B, there is a positive correlation between the number of cells in the desired state and an increment in noise intensity. When the copy number of DNA producing RNAi exceeded 5, the correlation became negative; namely, the increment of noise pushed cells away from the desired state. The distributions of P1 and P2, with the 5 copies of DNA producing RNAi, are shown in Fig 6C and 6D. The distributions of P1 and P2, with 30 copies of DNA producing RNAi, are shown in Fig 6E and 6F. A plausible explanation for the huge noise preventing cells from the ON state of P1 is that the substantial amount of noise widens the distribution and push P1 toward zero. The number of cells with P1 of zero in Fig 6C is 185, and that in Fig 6E is 326. In summary, the method of utilizing RNA-RNA interaction to drive cells to the desired state is inefficient and limited.

## Discussion

In this study, we illustrated that the internal noise can be utilized to instruct cells to the desired steady state. In reality, it is impossible to adjust the concentration of a stimulator to negative values and the addition of stimulator can drive cells toward only one direction. Remarkably, through manipulation of the noise intensity, cells can be driven to the other direction. Furthermore, bistability is the simplest type of multiple steady states; for systems with more than two stable steady states, it is somehow tough to use the stimulator to drive cells to a middle stable steady states. The method of utilizing internal stochasticity is possibly of use to guide cells to the desired state in such a situation.

**Fig 7:**
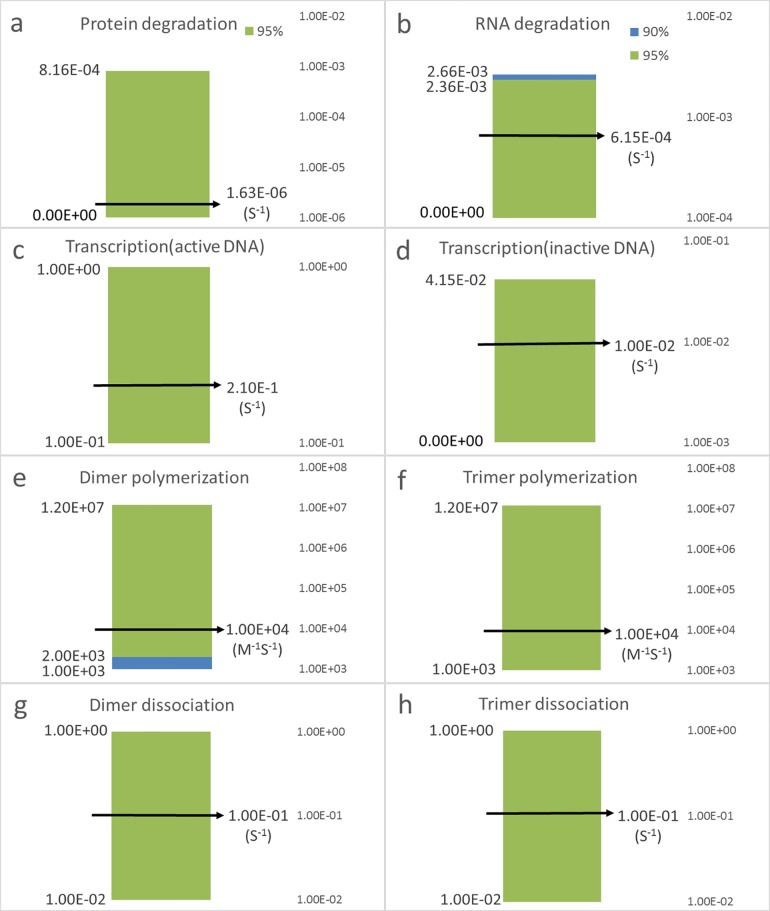
The sensitivity analysis showed the robustness of the method The value of each parameter was varied individually over a range while keeping all the other parameters at the nominal values, which is indicated by the arrow mark. We examined **a)** the rate constant of protein degradation, **b)** the rate constant of RNA degradation, **c)** the transcription rate from DNA in the active conformation, **d)** the transcription rate from DNA in the inactive conformation, **e)** the rate constant of forming protein dimer, and **f)** the rate constant of forming protein trimer. **g)** and **h)** are the dissociating rate constant of dimer and trimer, respectively. Remarkably, more than 95% of cells can be instructed, by internal noise, to the desired state for nearly all sets of parameter values (green bar in the figures). For the rest sets of parameter values, there are more than 90% of cells can be instructed, by internal noise, to the desired state (cyan bar in the figures).

We employed biologically practicable methods to adjust the stochasticity of gene expression. With careful selections of transcription and translation rates, different combinations provided the same protein level but revealed distinct stochastic nature. The adjustability of noise via translation rate was experimentally verified [29]. Such a genetic manipulation is not difficult to perform nowadays and the path leading to the outcome of Fig 4 is clear. It is also interesting to understand how the system behaves under different values of parameters. First, we changed the rate constant of protein degradation (both P1 and P2) from 1.63 x 10^−2^ (1/s) to 1.63 x 10^−6^ (1/s) because this value is more common for most of the systems. In order to keep the bistability, the translation rate was adjusted to 2.32 x 10^−6^ (1/s). With all other values of parameters in Table 4, the bistable curves remained similar to Fig 2A and 2B except for the scale of the x-axis (S6 Fig). We then searched for the parameter ranges of bistability by changing one parameter value at a time. Table 5 are the outcome for which bistability is observed. Note that the same parameters were applied to gene 1 and 2. We didn’t perform the sensitivity analysis for the full bistable parameter ranges because the deviant effect [30, 31] between deterministic and stochastic behaviors may just blow the bistability. Instead, the sensitivity analysis was conducted within a little smaller ranges of parameter values (Fig 7). The original bimodal distribution, for all sets of parameters, were 50% of P1 ON and 50% of P1 OFF due to following two reasons. First, the same parameter values were assigned to P1 and P2 so the number of P1 ON should equal to that of P2 ON. Second, for this mutually inhibitory system, the number of cells in P1 ON state should be equal to that of P2 OFF state. In brief, cells were equally distributed into ON state and OFF state of P1. We then applied the same method to manipulating internal noise. We kept stimulator of zero and the change of the internal noise drove cells to the desired state. Specifically, we adjusted the translation rate constant of P2 to 100 (1/s) but kept the protein levels the same by tuning the transcription rate of gene 2. Remarkably, more than 90% of cells were instructed to ON of P1 for all sets of parameter values (Fig 7). This outcome suggested that the presented method is not sensitive to the values of parameters. The minutiae of how much noise increased for each set of parameters are in Text E in S1 File.

**Table 5:** Ranges of parameter values for which bistability is observed.

**Rate constants**	**Nominal Values**	**Range of Bistability**		**Units**
		Min.	Max.	
Protein degradation, $k_{dP{}_{1}}$ = $k_{dP_{2}}$	1.63E-06	0.00E+00	9.17E-04	S^-1^
RNA degradation, $k_{dR{}_{1}}$ = $k_{dR{}_{2}}$	6.15E-04	0.00E+00	2.95E-03	S^-1^
Transcription from active DNA, $k_{RA{}_{1}}$ = $k_{RA{}_{2}}$	2.10E-01	7.19E-02	1.00E+01	S^-1^
Transcription from inactive DNA, $k_{RI{}_{1}}$ = $k_{RI{}_{2}}$	1.00E-02	0.00E+00	4.61E-02	S^-1^
Dimer polymerization, $k_{PP_{1}}$ = $k_{PP_{2}}$	1.00E+04	2.67E+02	1.33E+07	M^-1^S^-1^
Trimer polymerization, $k_{PPP_{1}}=k_{PPP_{2}}$	1.00E+04	2.67E+02	1.33E+07	M^-1^S^-1^
Dimer dissociation, $k_{-PP{}_{1}}$ = $k_{-PP_{2}}$	1.00E-01	7.51E-05	3.75E+00	S^-1^
Trimer dissociation, $k_{-PPP_{1}}$ = $k_{-PPP_{2}}$	1.00E-01	7.51E-05	3.75E+00	S^-1^

The influence from the addition of stimulator on the value of steady state is huge (Fig 2A). The ON state of P1 dropped to nearly half of the value at a stimulator concentration of 0.0113. This side effect can be easily overcome by the new method proposed in this study. The change of noise intensity has no influence on the deterministic behavior, both the steady state and dynamic behaviors. As for the averaged value from the stochastic model, it is also valid except for the situation that the bimodal distribution of P2 collapsed due to the high intensity of noise. Under such circumstances, the deviant effect [30, 31] could appear in the system and cause a shift of the averaged value. However, the direction of this shift is opposite to that caused by the addition of the stimulator.

Fig 1A shows the essential components leading to bistable responses, and the method demonstrated in this study showed a potential for application to a variety of systems shown in Fig 1B. In a comparison of Fig 1A, there are two major differences in Fig 1B. First, some systems in Fig 1B contain a positive feedback acting on its own promoter. Second, there is a heterodimer composed of both P1 and P2 in Fig 1B. With some modifications, these features can be incorporated into the presented model. In future work, the model will be extended to include the aforementioned features, thus enabling the presented method to be applied to many other systems. Most of the regulatory networks in Fig 1B are natural systems, and the control of the bimodal distribution for these systems is of great value.

For other methods presented in this work, although the method of utilizing RNA-RNA interaction showed less efficiency in driving cells to the desired state, this method can be applied by introducing a genetically engineered plasmid and is much easier to perform than the other two methods. Moreover, it is also possible to be applied by directly adding RNA to the mammalian cells without any gene modification.

One application of altering noise intensity to influence the bimodal distribution is the detection of latent HIV [32]. The increase in noise intensity caused by drug administration drove latent viral particles to be reactive and become detectable. The quiescent state is one of the major problems in HIV infection, and the state switch induced by noise is a novel solution. However, the current understanding of how to adjust the bimodal distribution remains insufficient. The increase in noise does not always favor the desired state; the system may favor the undesired state, as shown in Fig 6B and the S4 Fig. A comprehensive understanding of how noise affects the bimodal distribution can elucidate these applications.

## Supporting Information

S1 Fig The variance purely coming from the ratio of cells at ON or OFF state. (DOCX)

S2 Fig The bimodal distribution from initiate condition with all intracellular states at OFF of P1 (DOCX)

S3 Fig The distributions of mRNA (DOCX)

S4 Fig The figure of instructing cells to the ON state of P2 (DOCX)

S5 Fig The increment of noise does not always guide more cells to the desired state (DOCX)

S6 Fig The bistable curves for the degradation rate constant of protein as 1.63 x 10^−6^ (DOCX)

S1 File Supplementary Material (DOCX)
