# Supplementary figures and images for: Driving Cells to the Desired State in a Bimodal Distribution through Manipulation of Internal Noise with Biologically Practicable Approaches
Source: PLoS One. 2016 Dec 2;11(12):e0167563. doi: 10.1371/journal.pone.0167563 (PMC5135133; doi:10.1371/journal.pone.0167563)

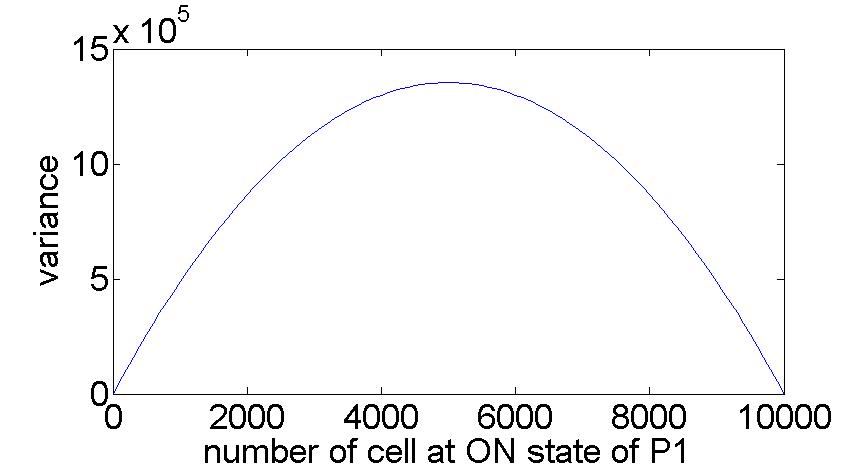


**S1 Fig** The variance purely coming from the ratio of cells at ON or OFF state.

Supplement: S1 Fig — (DOCX) [file pone.0167563.s001.docx]
