# Supplementary material for: Driving Cells to the Desired State in a Bimodal Distribution through Manipulation of Internal Noise with Biologically Practicable Approaches
Source: PLoS One. 2016 Dec 2;11(12):e0167563. doi: 10.1371/journal.pone.0167563 (PMC5135133; doi:10.1371/journal.pone.0167563)
Supplement: S2 Fig — (DOCX) [file pone.0167563.s002.docx]

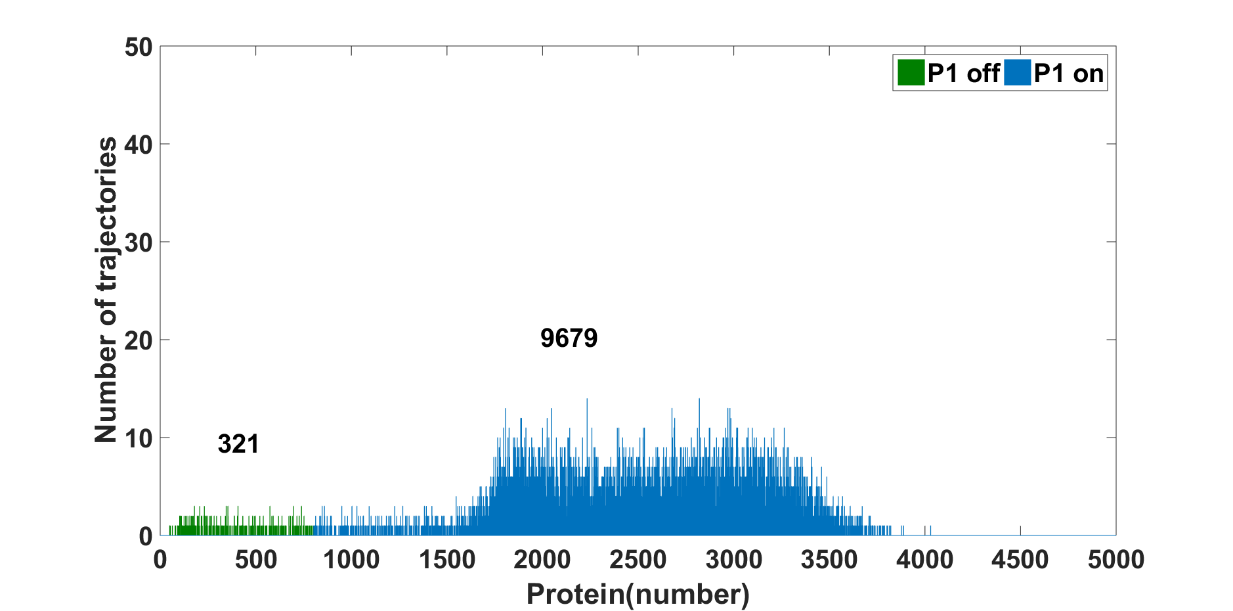


**S2 Fig** The bimodal distribution from initiate condition with all intracellular states at OFF of P1
All intracellular states are set to OFF stable steady state of P1 for the simulation; 9642 out of 10000 cells was driven to ON state of P1. Although the outcome is slightly different from Fig.4g, it is rational to believe that such small difference, only 0.3%, is due to computational error from the Stochastic Simulation Algorithm (SSA). This result supports that cells could be pushed from OFF state to ON state by manipulating the stochasticity.
