# Supplementary material for: Driving Cells to the Desired State in a Bimodal Distribution through Manipulation of Internal Noise with Biologically Practicable Approaches
Source: PLoS One. 2016 Dec 2;11(12):e0167563. doi: 10.1371/journal.pone.0167563 (PMC5135133; doi:10.1371/journal.pone.0167563)
Supplement: S3 Fig — (DOCX) [file pone.0167563.s003.docx]

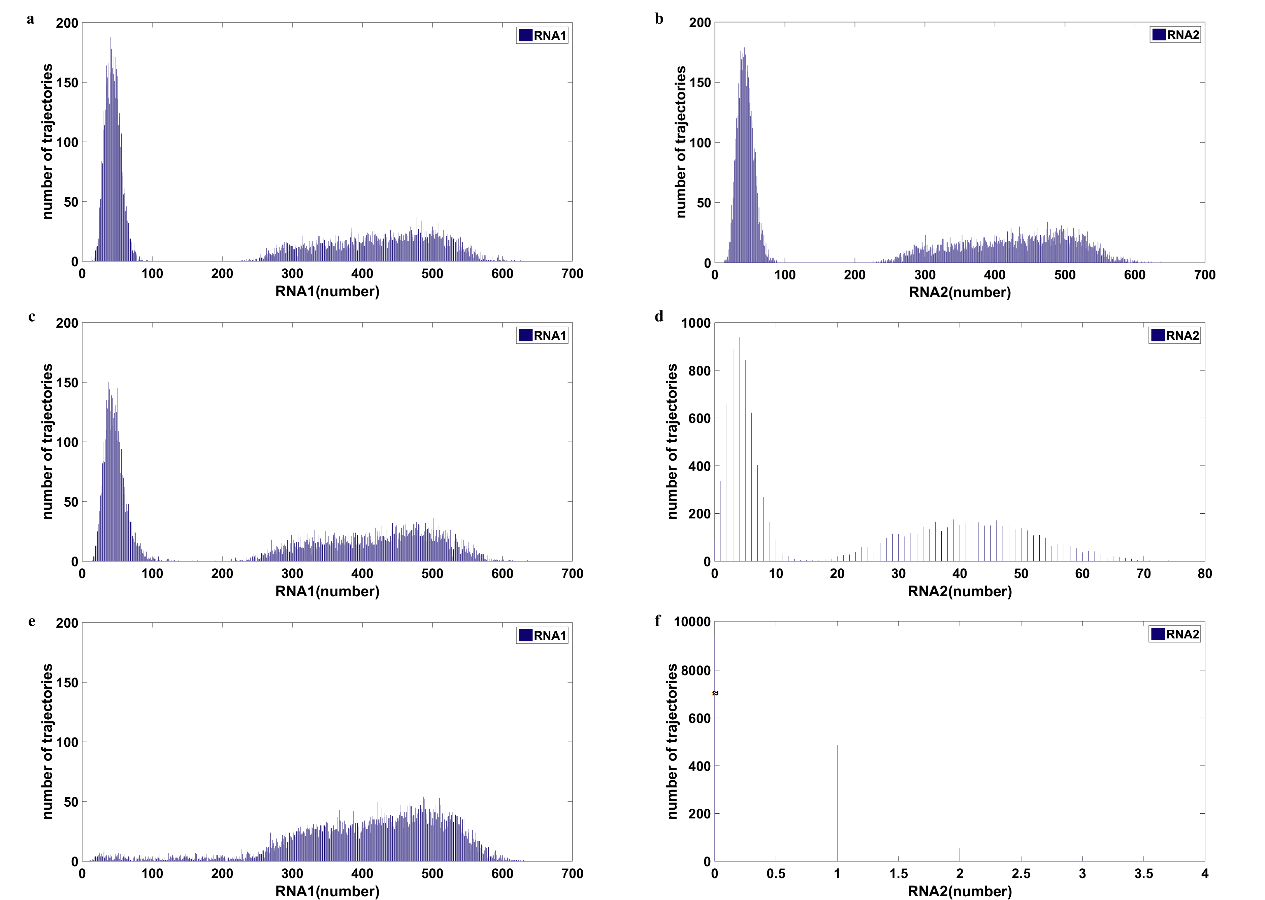


**S3 Fig** The distributions of mRNA

**a)** and **b)** are the distribution of RNA1 (the mRNA of gene 1) and RNA2 (the mRNA of gene 2), respectively, with the values of parameters listed in Table 4. **c)** and **d)** are the distributions of RNA 1 and RNA 2 with the translation rate of P2 increased to 1 (1/s). Note that there was only a slight change in the distribution of RNA1 though the distribution of RNA2 was dramatically shifted. **e)** and **f)** are the distribution of RNA 1 and RNA 2 with the translation rate of P2 further increased to 100 (1/s). The bimodal distribution was collapsed and most of the cells with RNA2 of zero.
