# Supplementary material for: Driving Cells to the Desired State in a Bimodal Distribution through Manipulation of Internal Noise with Biologically Practicable Approaches
Source: PLoS One. 2016 Dec 2;11(12):e0167563. doi: 10.1371/journal.pone.0167563 (PMC5135133; doi:10.1371/journal.pone.0167563)
Supplement: S4 Fig — (DOCX) [file pone.0167563.s004.docx]

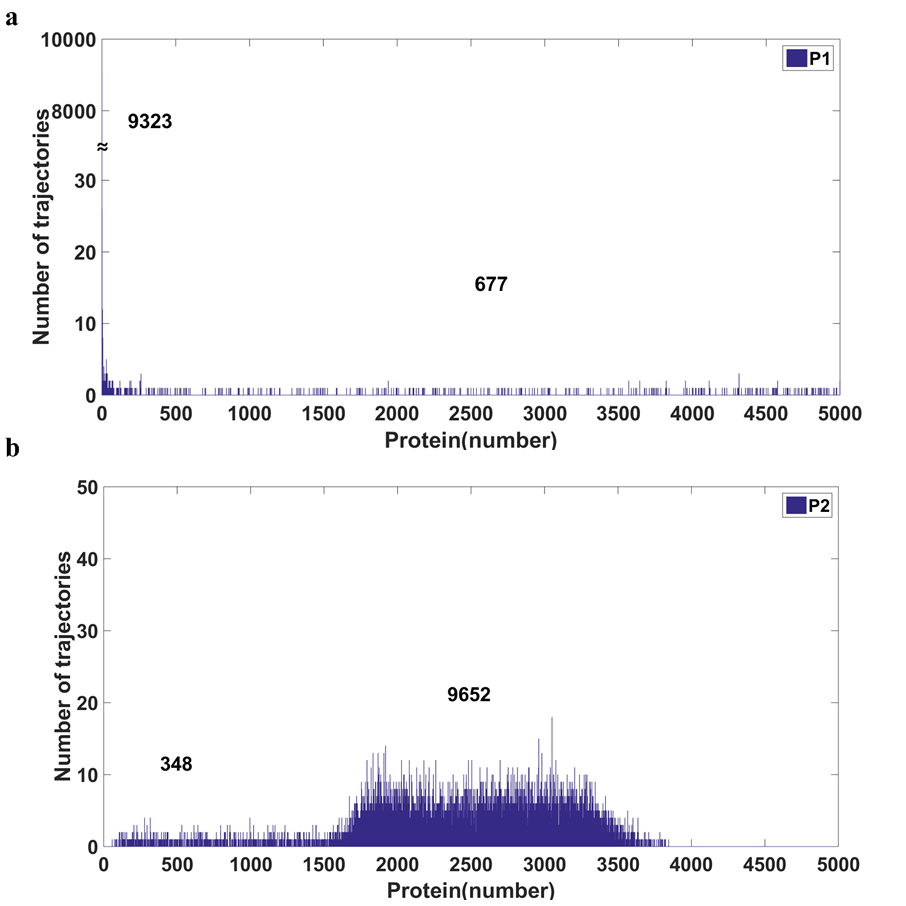


**S4** **Fig** The figure of instructing cells to the ON state of P2

**a)** and **b)** are the distribution of P1 and P2, respectively. With manipulation of translation rate of P1 equal to 100 (1/s), the cells have been guided to the ON state of P2.
