# Supplementary material for: Driving Cells to the Desired State in a Bimodal Distribution through Manipulation of Internal Noise with Biologically Practicable Approaches
Source: PLoS One. 2016 Dec 2;11(12):e0167563. doi: 10.1371/journal.pone.0167563 (PMC5135133; doi:10.1371/journal.pone.0167563)
Supplement: S5 Fig — (DOCX) [file pone.0167563.s005.docx]

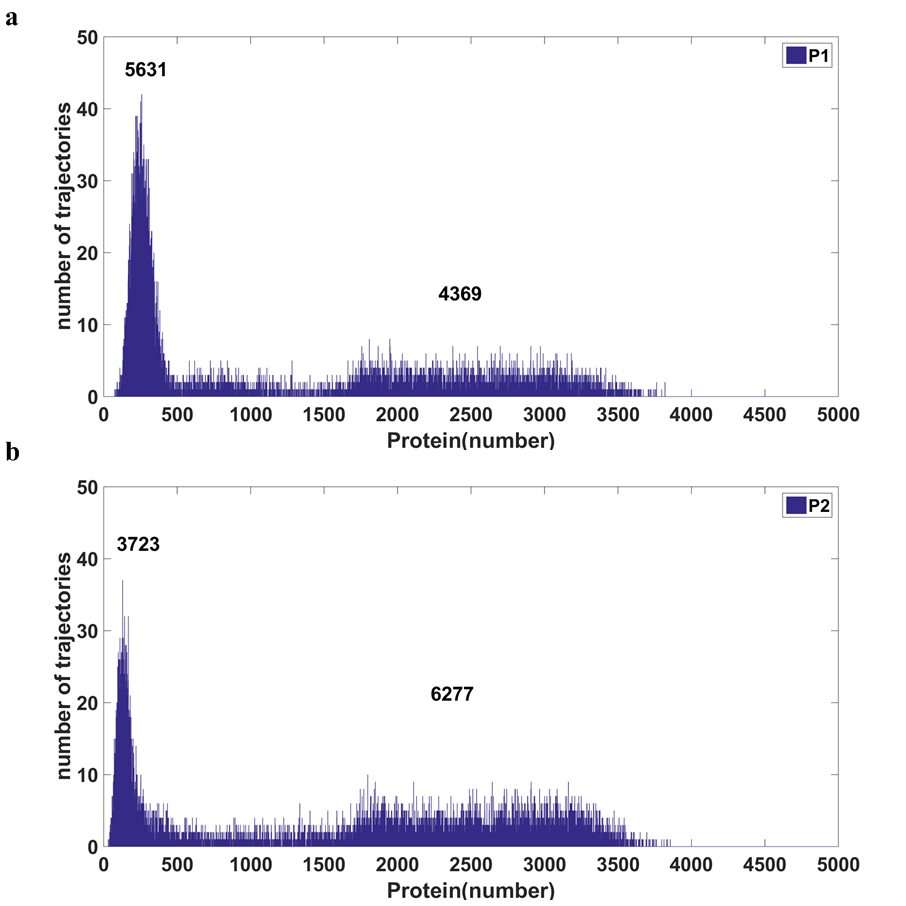


**S5** **Fig** The increment of noise does not always guide more cells to the desired state

**a)** and **b)** are the distribution of P1 and P2, respectively. When noise is further increased

with the rate constant of P1 trimer binding to DNA as 10^4^ (1/s M), cells migrated back to the OFF state of P1.
