# Supplementary material for: Driving Cells to the Desired State in a Bimodal Distribution through Manipulation of Internal Noise with Biologically Practicable Approaches
Source: PLoS One. 2016 Dec 2;11(12):e0167563. doi: 10.1371/journal.pone.0167563 (PMC5135133; doi:10.1371/journal.pone.0167563)
Supplement: S6 Fig — (DOCX) [file pone.0167563.s006.docx]

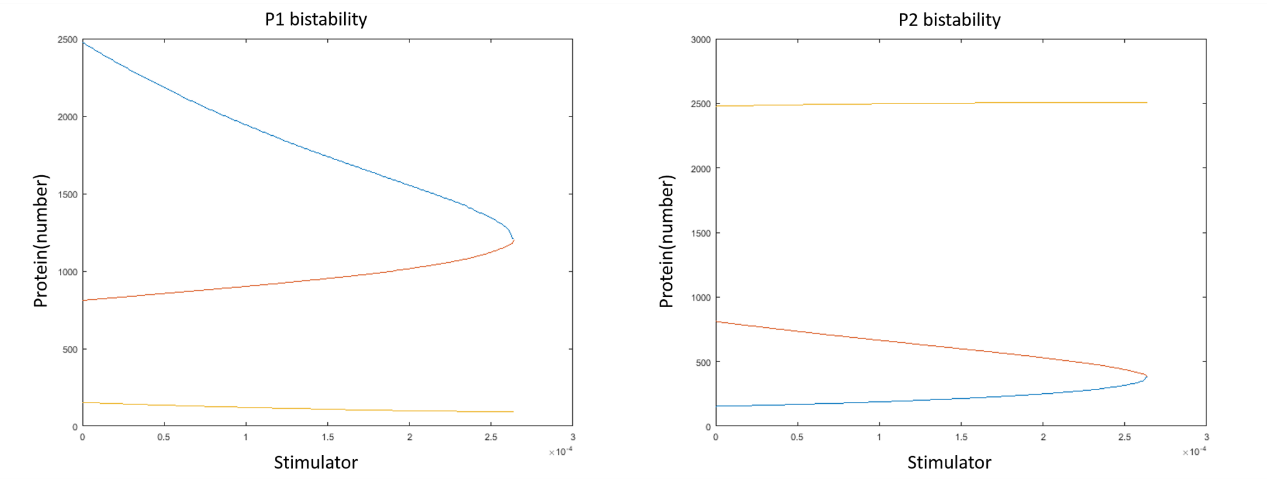


**S6 Fig** The bistable curves for the degradation rate constant of protein as 1.63 x 10^-6^

The bistable curves of P1 and P2, respectively. The x-axis represents the stimulator levels.
