# Supplementary material for: Driving Cells to the Desired State in a Bimodal Distribution through Manipulation of Internal Noise with Biologically Practicable Approaches
Source: PLoS One. 2016 Dec 2;11(12):e0167563. doi: 10.1371/journal.pone.0167563 (PMC5135133; doi:10.1371/journal.pone.0167563)
Supplement: S1 File — (DOCX) [file pone.0167563.s007.docx]

S1 File
Supplementary Material

**Driving Cells to the Desired State in a Bimodal Distribution through Manipulation of Internal Noise with Biologically Practicable Approaches**

Che-Chi Shu*,Chen-Chao Yeh, Wun-Sin Jhang, Shih-Chiang Lo

**Text A**

We adapted parameters from two articles. One is Tian et al. (the Ref -5 in the main text) and the other is Mehra et al. (the Ref -25 in the main text). The $k_{RA _{1}}$and $k_{RA _{2}}$ (transcription rate constants of active gene 1 and gene 2, respectively) were 2.1x 10^-1^ which were picked within the ranges of 1.8x 10^-1^ and 4.5x 10^-1^, the rate constants of scbR mRNA transcription and scbA mRNA transcription in Mehra’s work. The $k_{P _{1}}$and $k_{P_{2}}$(translation rate constants) were 10^-1^ which was picked within the ranges of 6.6x 10^-2^ and 3.6x 10^-1^, the rate constants of ScbA translation and ScbR translation in Mehra’s work. The $k_{DI_{2}}$and $k_{-DI _{2}}$were chosen to have the equilibrium constant of 10 because the equilibrium constant for binding of ScbR to O_R_ in Mehra’s work is 8.82. Similarly, we have $k_{DI _{ 1}}$ and $k_{-DI _{ 1}}$.

The production rate from inactive DNA is only 1/21 of that from active DNA, the α to (α+β) in Tian’s work. Applied this to our work, we have $k_{RI _{ 1}}$ and $k_{RI _{2}}$as 10^-2^ because the transcription rate constants of active DNA is 2.1x 10^-1^ in our study. The

$k_{dR _{1}}$and $k_{dR _{2}}$were adapted from α in Tian’s work. If we convert α to our study, α =$k_{P _{1}}$[*R*_1 from basal expression_ ] and [*R*_1 from basal expression_] = ($\frac{k_{{RI}_{1}}}{k_{{dR}_{1}}+\mu}$)([*DNA*_total_]) where the unit of [*DNA*_total_] is concentration instead of copy number and its value is 3.3 nM. By substituting $k_{{RI}_{1}}$, µ and [*DNA*_total_] into the equations, the $k_{dR _{1}}$was determined. Similarly, the$k_{dR _{2}}$ can be determined. The degradation rate of protein and the rate constants relating to protein monomer to trimer were chosen in order to have bistability.

**Text B**

We conducted the simulations for 30000s and 60000s for the case of Fig 4g. The outcome of P1 is following.

|  | 30000s | 60000s |
| --- | --- | --- |
| mean | 2459.21 | 2462.58 |
| CV | 0.2813 | 0.2800 |

**Text C**

We will use Eq. (3)-(7) of Table 2 to illustrate how to manipulate the noise of P1 protein without altering its deterministic value. A similar method could be applied to P2 protein with Eq. (1) and (9)-(12) of Table 2. The steady state equations for Eq. (3)-(7) of Table 2 are following equations.

k_−DI2_ [*DI*_2_] – k _DI2_ [*PPP*_1_][*DA*_2_] = 0 (S1)

k_PPP1_[*PP*_1_][*P*_1_] −k_-PPP_1[*PPP*_1_] −k_DI2_ [*PPP*_1_][*DA*_2_]+k_−DI2_[*DI*2] = 0 (S2)

k_PP1_[*P*_1_]^2^−k_−PP1_[*PP*_1_] − k_PPP1_[*PP*_1_][*P*_1_]+k_−PPP1_[*PPP*_1_] = 0 (S3)

k_P1_[*R*_1_] −2k_PP1_[*P*_1_]^2^+2k_−PP1_[*PP*_1_] –k_PPP1_[*PP*_1_][*P*_1_]+k_−PPP1_[*PPP*_1_] –(k_dP1_+μ+k_r_)[*P*_1_] = 0 (S4)

When we substitute Eq.(S1) into Eq.(S2), Eq.(S2) is simplified.
k_PPP1_[*PP*_1_][*P*_1_] −k_-PPP_1[*PPP*_1_] = 0 (S5)

Next, we substituted Eq.(S5) into Eq.(S3).
k_PP1_[*P*_1_]^2^−k_−PP1_[*PP*_1_]=0 (S6)

Finally we substituted Eq. (S5) and (S6) into Eq.(S4).

k_P1_[*R*_1_] –(k_dP1_+μ+k_r_)[*P*_1_] = 0 (S7)

The Eq.(S7) shows the relationship between protein level and RNA level. If we could further acquire information of DNA configuration to RNA level, we could link protein level to DNA configuration. The steady state of Eq.(3) in Table 2 gives the information of DNA configuration to RNA level.
[*R*_1_] = ($\frac{k_{{RA}_{1}}}{k_{{dR}_{1}}+\mu}$)([*DA*_1_] +($\frac{k_{{RI}_{1}}}{k_{{RA}_{1}}}$)[*DI*_1_]) (S8)

The combination of Eq. (S7) and (S8) gives us the information of protein level to DNA configuration.
[*P*_1_] =$\left( \frac{k_{P1}}{k_{dP1}+\mu+k_{r}} \right)$($\frac{k_{{RA}_{1}}}{k_{{dR}_{1}}+\mu}$)([*DA*_1_] +($\frac{k_{{RI}_{1}}}{k_{{RA}_{1}}}$)[*DI*_1_]) (S9)

We keep all other rate constant, except $k_{P1},k_{{RA}_{1}} and k_{{RI}_{1}}$, the same so the Eq.(S9) could be further simplified to following equation.

[*P*_1_] =constant$\times\left( k_{P1} \right)$($k_{{RA}_{1}}$)([*DA*_1_] +($\frac{k_{{RI}_{1}}}{k_{{RA}_{1}}}$)[*DI*_1_]) (S10)

In order to keep the protein level, we need to make sure the value of product $\left( k_{P1} \right)$($k_{{RA}_{1}}$) as well as the fraction ($\frac{k_{{RI}_{1}}}{k_{{RA}_{1}}}$) the same for each case.

**Text D**

Here, we explain why the maximum value of variance would happen while cells were equally divided into two models. Two subpopulations are introduced, with one representing cells at the OFF state of P1 and the other at ON state. We forced zero noise to each subpopulation. Namely, for subpopulation with OFF state of P1, all cells are with P1 at 153.5 as shown in Fig 2a; similar, for subpopulation with ON state of P1, all cells are with P1 at 2480. Clearly, the variance is zero for each subpopulation because cells are exactly the same within the same subpopulation. Intriguingly, the variance of the whole population in not zero but depends on the ratio of cells at ON or OFF state. To better illustrate this concept, we have plotted the variance verse the ratio of cells at ON state (S1 Fig.); when cells are equally distributed to each mode, the variance is 1.353E6. Comparing this value to the variance of Fig 2c, 1.449E6, less than 7% of variance is from the distribution within the subpopulation

**Text E**

It is, of course, the internal noise increased if we increased the translation rate constant of P2 to 100 (1/s) but keep the P2 protein level untouched by tuning the transcription rate constant of gene 2. It notably influenced the coefficient of variation (COV) of P2 OFF state. This phenomenon was illustrated in the Fig 3e. We listed the coefficient of variation of P2 OFF state in the following table for different parameter values. The second row of the table indicates the COV change for nominal values of parameters. The rest rows are the value of each parameter varied individually while keeping all the other parameters untouched.

| Parameter | Value | COV (before adjusting the translation rate constant of P2) | COV (after adjusting the translation rate constant of P2 to 100 S^-1^) |
| --- | --- | --- | --- |
| with nominal parameter values |  | 0.237194 | 9.949874 |
| $k_{dP _{1}}$= $k_{dP_{2}}$= | 8.16E-04($S^{-1}$) | 0.055767 | 9.511769 |
|  | 0.00E+00($S^{-1}$) | 0.186506 | 9.848858 |
| $k_{dR _{1}}$= $k_{dR _{2}}$= | 2.66E-03($S^{-1}$) | 0.057569 | 5.833918 |
|  | 2.36E-03($S^{-1}$) | 0.055206 | 9.051966 |
|  | 0.00E+00($S^{-1}$) | 0.268153 | 9.949874 |
| $k_{RA _{1}}$= $k_{RA _{2}}$= | 1.00E+00($S^{-1}$) | 0.244918 | 9.445881 |
|  | 1.00E-01($S^{-1}$) | 0.107697 | 9.546959 |
| $k_{RI _{ 1}}$= $k_{RI _{2}}$= | 4.15E-02($S^{-1}$) | 0.162968 | 9.848857 |
|  | 0.00E+00($S^{-1}$) | 0.270338 | 9.949874 |
| $k_{PP_{ 1}}$= $k_{PP_{2}}$= | 1.20E+07($M^{-1}S^{-1}$) | 0.053916 | 12.84114 |
|  | 2.00E+03($M^{-1}S^{-1}$) | 0.220993 | 9.797959 |
|  | 1.00E+03($M^{-1}S^{-1}$) | 0.098956 | 6.509011 |
| $k_{PPP_{ 1}}$= $k_{PPP_{2}}$= | 1.20E+07($M^{-1}S^{-1}$) | 0.098533 | 9.899495 |
|  | 1.00E+03($M^{-1}S^{-1}$) | 0.096714 | 9.825525 |
| $k_{-PP _{1}}$= $k_{-PP_{2}}$= | 1.00E+00($S^{-1}$) | 0.095934 | 9.777403 |
|  | 1.00E-02($S^{-1}$) | 0.427728 | 9.795854 |
| $k_{-PPP_{ 1}}$= $k_{-PPP_{2}}$= | 1.00E+00($S^{-1}$) | 0.072224 | 9.576991 |
|  | 1.00E-02($S^{-1}$) | 0.244990 | 9.899494 |
